# Lightweight deep learning for tomato disease detection: trends, challenges, and edge AI perspectives

**DOI:** 10.3389/fpls.2025.1737208

**Published:** 2026-02-12

**Authors:** Harshinisree Gunasekaran, Sujatha Rajkumar, Lincy Kirubhadharsini B.

**Affiliations:** 1 School of Biosciences and Technology (SBST), Vellore Institute of Technology (VIT), Vellore, India; 2 VIT School of Electronics Engineering (SENSE), Vellore Institute of Technology (VIT), Vellore, India; 3 VIT School of Agricultural Innovations and Advanced Learning (VAIAL), Vellore Institute of Technology (VIT), Vellore, India

**Keywords:** deep learning, edge AI, insect vectored disease, lightweight models, tomato leaf disease, transformer models, uncertainty quantification

## Abstract

Tomato (*Solanum lycopersicum*) is a globally cultivated horticultural crop, yet its productivity is severely constrained by foliar and insect-vectored diseases that reduce its quality and production. Early and accurate diagnosis of these diseases, along with sustainable biocontrol strategies, is essential for improving crop health and reducing economic losses. This review synthesizes and evaluates the recent progress in lightweight deep learning models and edge AI for tomato disease detection, highlighting their potential for practical deployment in precision agriculture. A comprehensive survey of recent literature was conducted, which covers convolutional neural networks, transformer-based models, optimization techniques including pruning, quantization, and knowledge distillation, and use of explainable AI tools to enhance transparency and trust. In addition, experimental validation was performed by utilizing MobileNetV2 and EfficientNetB0 on a subset of tomato diseases that are most common and prevalent in Tamil Nadu. The test performance of both the models resulted in an overall accuracy of 99.9% and macro-F1 nearly 0.99. Further, a unique framework that combines AI-powered diagnosis with microbial biocontrol recommendations is proposed offering a solution to manage diseases in both eco-friendly and region-specific way. Overall, this work provides a roadmap for combining sustainable methods with AI-driven diagnosis, promoting resilient, scalable, and farmer-friendly agricultural systems.

## 1 Introduction

Tomato (*Solanum lycopersicum*) is a significant agricultural crop that is extensively grown for its nutritional value, economic significance and use in both fresh and processed food systems. It is an essential crop to rural livelihoods and food security particularly in nations like India and it grows across a variety of agro-climatic zones (Mohanty et al., 2016). Although there are improvements in agronomic practices and input management, tomato production is hindered by a variety of foliar and insect-vectored diseases such as early blight (*Alternaria solani*), bacterial spot (*Xanthomonas* spp.), septoria leaf spot (*Septoria lycopersici*), and tomato yellow leaf curl virus (TYLCV) (Durmuş et al., 2017; Fuentes et al., 2017).

Changing climatic patterns, monoculture systems and the increased resistance of pests and pathogens to chemical controls, contributes to improved frequency and severity of these diseases (Ma et al., 2021; Bebber et al., 2013; Garrett et al., 2006). In countries like India, millions of smallholder farmers practise tomato farming, where delayed disease detection often results in substantial yield losses, reduced fruit quality and unforeseen financial difficulties.

Earlier, disease diagnosis often depends on manual inspection by experienced personnel, that is labor-intensive, time-consuming and mostly inconsistent in field conditions. Also, the lack of trained professionals in rural and remote areas, resulted in overuse of agrochemicals as well as delayed or inaccurate disease diagnosis (Ahmed et al., 2022). Despite the continued usage of chemical pesticides, their environmental risks and decreasing effectiveness due to resistance have forced to look for more sustainable and intelligent solutions (Pretty and Bharucha, 2015).

Deep Learning (DL) has become a breakthrough in plant disease diagnostics over the last ten years, allowing for the automated, highly accurate diagnosis of leaf symptoms from digital images. A broad range of tomato diseases have been classified using Convolutional neural networks (CNNs) like ResNet, VGG and MobileNet (Ferentinos, 2018; Too et al., 2019). In recent years, vision transformers (ViTs) and hybrid models has gained attention due to their ability to extract global feature representations and long-range dependencies resulting in classification robustness under variable conditions (Nishankar et al., 2025; Alshammari, 2024).

However, the computational demands of these models are a major obstacle to the widespread adoption of these models. Most high-performing DL models are optimized for powerful GPUs or cloud environments, which are often inaccessible in the field (Howard et al., 2017). This has led to a shift in focus toward lightweight architectures and optimization strategies such as pruning (removing unnecessary model parameters to reduce size and speed up inference), quantization (reducing the numerical precision of model parameters to speed up inference on edge devices) and knowledge distillation (a method where a smaller model learns from a larger, high-capacity model to improve efficiency) that enable real-time inference on low-power, embedded edge devices like smartphones, Raspberry Pi and NVIDIA Jetson Nano (Han et al., 2016; Howard et al., 2017).

In Indian agriculture, edge AI-based diagnostic tools has immense potential. Their offline operation, portability and affordability make them ideal for use in rural farming communities where the Internet connectivity is inconsistent and infrastructure is minimal. The opportunity to make these tools available as mobile applications tailored in local languages can help farmers get the right information at the right time to make timely decisions.

Even with these significant advancements, current AI models often suffer from dataset imbalance, image variability, poor generalization across field environments, and overlapping visual symptoms. According to Mustofa et al., 2023, most benchmark datasets utilized for training have low diversity and are frequently recorded in controlled environment, which restricts their application in real-world. As a result, gathering field representative datasets and validating models in real-world settings are increasingly important.

Furthermore, as these deep learning models are involved in important agricultural decisions, their successful adoption depends on their transparency and adaptability, so that farmers can trust and interpret its decisions. Researchers and practitioners may visualize model attention and determine which region of the leaf influences predictions with tools like Grad-CAM and SHAP, increasing model transparency and user trust (Selvaraju et al., 2017; Lundberg and Lee, 2017).

Integration of AI diagnostics with microbial biocontrol strategies is another crucial part but unexplored aspect of tomato disease management. Beneficial microorganisms including *Bacillus subtilis*, *Trichoderma* spp., and *Beauveria bassiana* have shown great potential in suppressing plant pathogens and insect vectors while boosting plant resistance (Chowdhury et al., 2015; Lacey et al., 2015). Eco-friendly and closed loop disease management, especially in sustainable and organic farming systems could be supported by combining real-time disease detection with targeted biocontrol applications.

Furthermore, recent studies indicates a connection between the dynamics of pest infestation and plant brix levels or sugar content. This link shows the possibility of integrated monitoring frameworks that assist early warning systems and proactive biocontrol deployment by combining plant physiological indicators with image-based disease identification.

This review intends to address the important gaps and opportunities in AI-based tomato disease detection through the following objectives:

- To provide a detailed analysis of lightweight deep learning architectures developed for tomato disease diagnosis.
- To offer a comparative overview of CNNs and transformer-based models in plant pathology.
- To outline and analyze the benchmark datasets commonly used in tomato disease detection research.
- To evaluate the practical feasibility and potential of edge AI deployments for in-field diagnosis.
- To integrate perspectives on AI-driven diagnostics with microbial biocontrol strategies for sustainable disease management.
- To highlight future research directions for the development of robust, scalable and farmer-accessible diagnostic tools tailored to tomato crop health monitoring.

**Figure 1:**
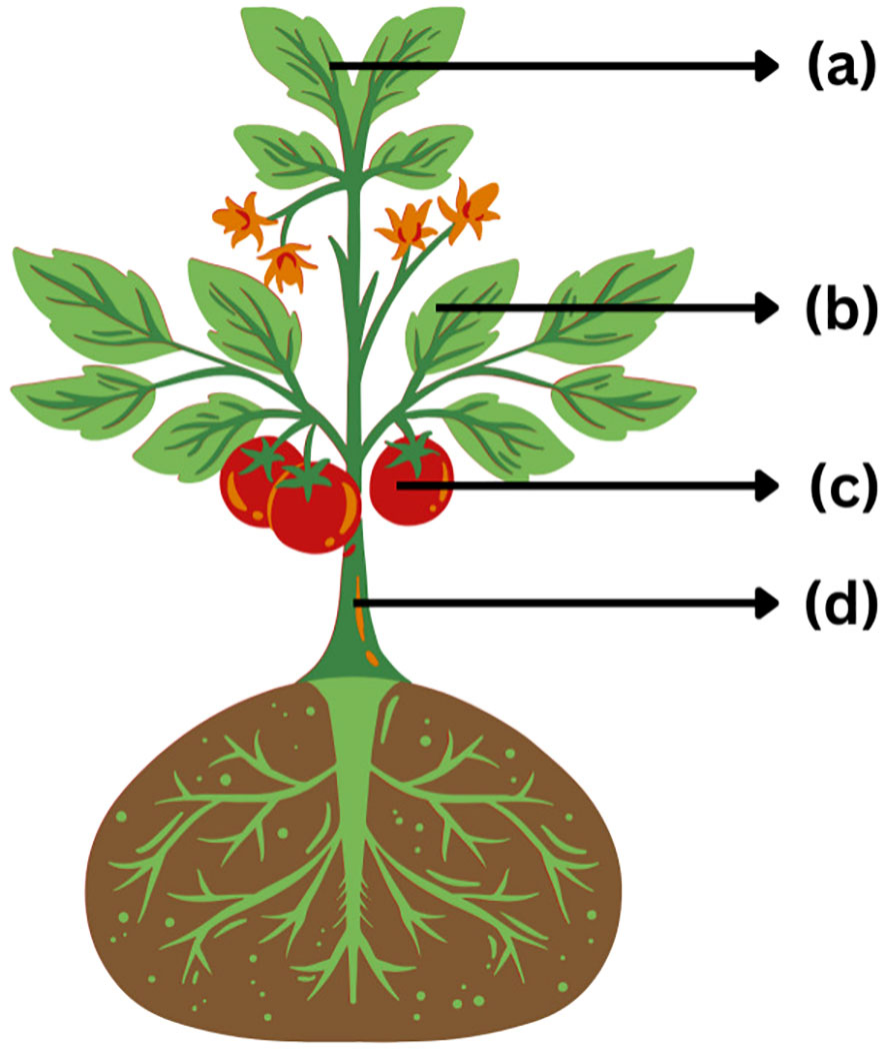
Schematic diagram of the tomato plant showing disease-prone regions by type of pathogen. **(a)** Young apical leaves are common entry points for insect-vectored viral infections such as TYLCV and TSWV. **(b)** Leaves are primary targets of fungal pathogens (e.g., early blight, septoria) and bacterial (e.g., bacterial spot, speck) diseases. **(c)** Fruits are affected by secondary fungal and bacterial pathogens. **(d)** Stems and vascular tissues are particularly susceptible to bacterial wilt.

**Figure 1** shows the structure of the tomato plant and the main areas affected by foliar pathogens and insect vectors.

## 2 Background

Tomato (*Solanum lycopersicum*) is cultivated worldwide and holds considerable economic importance globally due to its high nutritional content and versatility in both fresh and processed forms. However, tomato cultivation is frequently challenged by a variety of diseases that affect various plant organs, including leaves, stems, and fruits. These diseases, are mostly triggered by fungal, bacterial, and viral pathogens, which results in severe yield losses, decreased fruit quality and significant financial losses for farmers.

Global agricultural estimates indicate that tomato crop diseases cause an annual production loss of up to 30%, with economic damages amounting to billions of dollars globally (FAO, 2021). Outbreaks of devastating diseases such as late blight and TYLCV can result in complete crop failure in affected regions. In addition to yield loss, indirect factors like reduced fruit quality, increased pesticide reliance, added labor, and regulatory hurdles significantly increase the financial pressure on both smallholder and commercial farmers.

### 2.1 Major disease categories and their impact

Tomato diseases can be broadly categorized according to their causal organisms such as fungal, bacterial and insect-transmitted viral pathogens. Fungal diseases such as early blight (*Alternaria solani*), late blight (*Phytophthora infestans*), septoria leaf spot (*Septoria lycopersici*), leaf mold (*Fulvia fulva*), and Fusarium wilt (*Fusarium oxysporum* f. sp. *lycopersici*) primarily affect the foliage and vascular systems, leading to defoliation, wilting and fruit rot (Jones et al., 2014; Nowicki et al., 2012).

Bacterial infections including bacterial spot (*Xanthomonas* spp.), bacterial speck (*Pseudomonas syringae*), bacterial canker (*Clavibacter michiganensis*), and bacterial wilt (*Ralstonia solanacearum*) disrupt photosynthesis, affects the vascular flow, and reduce overall plant vigor (Hayward, 1991; Timilsina et al., 2020). Insect-vectored viral diseases, such as Tomato Yellow Leaf Curl Virus (TYLCV), Tomato Spotted Wilt Virus (TSWV), and Tomato Chlorosis Virus (ToCV), spread rapidly through vectors like whiteflies and thrips, causing symptoms like leaf curling, interveinal chlorosis, and stunted growth (Hanssen et al., 2010; Lapidot and Friedmann, 2002).

**Figure 2:**
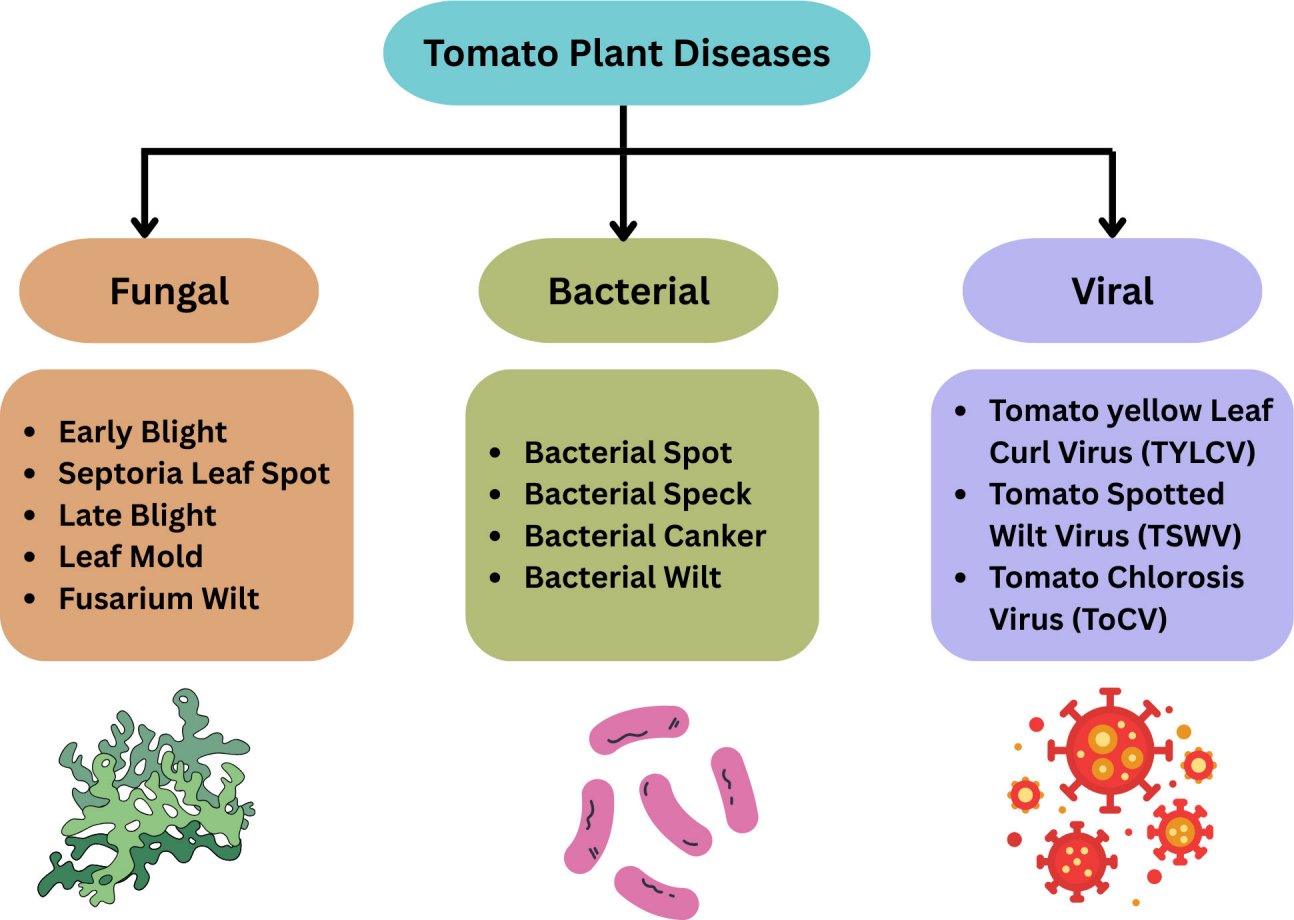
Major tomato plant diseases categorized by type of pathogen.

**Figure 2** shows the visual categorization of tomato plant diseases and **Table 1** summarizes their symptoms, mode of transmission and geographical relevance.

**Table 1:** Summary of major tomato plant diseases.

Disease	Causal organism	Category	Key symptoms	Transmission	Geographical impact
Early Blight	*Alternaria solani*	Fungal	Concentric brown spots on lower leaves; defoliation	Airborne spores	Global, especially warm regions
Late Blight	*Phytophthora infestans*	Oomycete (fungal-like)	Water-soaked lesions with white mold underside	Air and rain-borne spores	Cool, moist areas
Septoria Leaf Spot	*Septoria lycopersici*	Fungal	Numerous small, gray-centered leaf spots	Rain splash, wind	Humid climates worldwide
Leaf Mold	*Fulvia fulva*	Fungal	Yellow patches, olive mold on leaf underside	Wind-dispersed spores	Greenhouse and humid regions
Fusarium Wilt	*Fusarium oxysporum* f. sp. *lycopersici*	Fungal	Yellowing, wilting of lower leaves; vascular browning	Soil-borne	Warm tropics and subtropics
Bacterial Spot	*Xanthomonas* spp.	Bacterial	Brown lesions on leaves, fruits; leaf drop	Splashing water, tools	Worldwide
Bacterial Speck	*Pseudomonas syringae* pv. *tomato*	Bacterial	Tiny black spots on fruit and foliage	Rain splash, seed-borne	Global
Bacterial Canker	*Clavibacter michiganensis* subsp. *michiganensis*	Bacterial	Wilting, edge burn, cankers on stems	Seed-borne, wounds	North America, Europe
Bacterial Wilt	*Ralstonia solanacearum*	Bacterial	Sudden wilting, vascular browning, bacterial ooze	Soil and water	Asia, Africa, tropics
TYLCV	Tomato Yellow Leaf Curl Virus	Viral	Leaf curling, yellowing, stunting	Whitefly vector	Asia, Africa, Americas
TSWV	Tomato Spotted Wilt Virus	Viral	Necrotic spots, bronzing, ring spots	Thrips vector	Worldwide
ToCV	Tomato Chlorosis Virus	Viral	Interveinal chlorosis, leaf thickening	Whitefly vector	Europe, Americas, Asia

Disease distribution patterns are significantly influenced by regional climatic conditions and crop management practices. For example, fungal diseases like early blight and septoria flourish in temperate and humid conditions whereas in tropical and sub-tropical zones, bacterial wilt and TYLCV will be high due to excessive humidity and vector activity. The alter in pathogen life cycles and extended vector habitats due to climate change, also contributes to the onset of diseases (Garrett et al., 2006).

### 2.2 Limitations of traditional disease management

Manual scouting, visual symptom evaluation and laboratory-based confirmation using PCR, ELISA or other culturing techniques are the primary approaches in traditional disease diagnosis. Due to the subjective nature of human observation and the similarity of symptoms across diseases, these methods are labor-intensive, time-consuming and are often incorrect (Bock et al., 2010). Moreover, many rural and low-resourse agricultural areas lack access to proper diagnostic labs and plant pathology experts.

And for quick disease suppression, farmers always turn to chemical pesticides. But, excessive and improper use of agrochemicals has increased pest resistance and contributed to environmental pollution, and consumers demand for chemical-free alternatives is still growing (Pimentel and Burgess, 2014). Climate variability complicates the situation even more, by altering the pathogen life cycles and insect vector dynamics, making disease outbreaks more unpredictable.

The tomato’s widespread cultivation, rich image-based symptom manifestation and economic significance has made it a model crop for AI-based plant disease diagnosis. Additionally, a number of publicly accessible annotated datasets like PlantVillage, Tomato Leaf Disease Dataset, and TDDS provide standardized resources for training and benchmarking deep learning models, making tomato a perfect model system for assessing AI methods in agriculture.

### 2.3 Rise of deep learning in plant pathology

Recent advances artificial intelligence (AI), especially deep learning (DL), has revolutionized disease diagnosis in the agriculture industry. Convolutional Neural Networks (CNNs), in particular have been widely utilized to accurately identify and categorize plant diseases from leaf images with high accuracy (Mohanty et al., 2016; Ferentinos, 2018). These models are useful tools for automating plant health monitoring, because they can recognize intricate visual patterns including color, texture and lesion shape.

Despite their popularity, traditional deep learning models sometimes require powerful GPUs and cloud-based servers due to their high processing demands. Their adoption in field settings is constrained by these limitations, especially in locations with limited resources (Kamilaris and Prenafeta-Boldú, 2018). As a result, research interest in lightweight architectures such as MobileNet, EfficientNet-Lite and SqueezeNet has increased.

### 2.4 Edge AI and lightweight deep learning

Edge AI is deploying AI models directly on devices such as smartphones, drones, or microcontrollers (e.g., Raspberry Pi, Jetson Nano) without the need for the Internet or cloud access. Edge AI in agriculture, facilitates faster disease detection, minimizes need on cloud connectivity and continues to be useful even in remote farming areas. To support edge deployment, researchers are applying model compression techniques such as pruning, quantization and knowledge distillation to reduce the model size and energy demand while retaining performance (Han et al., 2016; Cheng et al., 2017). Transformer-based models like ViT (Vision Transformer) are also being adapted to lightweight formats for plant disease diagnosis (Dosovitskiy et al., 2021).

### 2.5 Microbial biocontrol as a sustainable strategy

In parallel with advances in AI innovations, microbial biocontrol has gained attention as a sustainable alternative to chemical pesticides. Beneficial microorganisms such as *Bacillus subtilis*, *Trichoderma harzianum* and *Beauveria bassiana* provide defense mechanisms that includes:

- Antibiosis
- Competing for space and nutrition
- Activation of plant immunological responses.
- Direct pathogen or insect vector parasitism (Guzmán-Guzmán et al, 2023; Benítez et al., 2004)

According to field studies, microbial biocontrol agents can improve plant growth, minimize disease severity and reduce the reliance on synthetic chemicals (Agha et al., 2023; Jiao et al., 2021). AI-powered early detection systems are a useful addition to existing systems but their efficacy primarily depends on timely and accurate application. The development of integrated, field-deployable solutions (addressed in the next section) is made possible by the integration of biological control strategies and AI-based disease diagnosis, which together offer a strong and long-lasting framework for precision crop protection.

## 3 Recent advances in lightweight deep learning for tomato disease detection

### 3.1 Deep learning architectures for tomato disease detection

Recent developments in deep learning (DL) have greatly enhanced computer vision, and agricultural diagnostics, especially in crop disease identification. Among these, tomato disease detection has become an important area of study due to the availability of annotated datasets, visual symptom distinctiveness and the economic importance of the crop. Deep learning models particularly Convolutional Neural Networks (CNNs) and currently, Vision Transformers (ViTs) had shown superior performance in image-based plant pathology tasks, including tomato leaf disease classification, severity estimation and lesion segmentation.

#### 3.1.1 Publicly available datasets

The efficacy of deep learning models for tomato disease identification highly depends on the quality, diversity and annotation accuracy of the training datasets. Most models are trained on image-based datasets containing leaf samples captured in controlled environment. However, issues such as dataset bias, class imbalance and limited representation of real-field conditions remain as major challenges. The commonly used datasets for tomato disease diagnoses is listed in **Table 2**.

**Table 2:** List of benchmark datasets for tomato leaf disease diagnoses.

Dataset name	Source/ platform	Tomato classes	Total tomato leaf images	Diseases covered	Notes
PlantVillage	Hughes and Salathé, 2015	10	5,868	Early blight, late blight, bacterial spot, mosaic, etc.	Publicly cited, lab+greenhouse
Tomato Leaf Disease Dataset	Kaggle	10	15,125	Includes blight, mold, virus, bacterial spotted varieties	Standard benchmark
Mendeley Tomato Dataset	Mendeley Data	9	10,000	Commercial/casual field images of fungal and viral diseases	Includes background noise support
AI Challenger Agriculture	AI Challenger competition data (2018) (China)	12	25,000+	Multiple crops; sub-set has tomato diseases	Challenging real-scene complexity

#### 3.1.2 Convolutional neural networks

**Figure 3:**
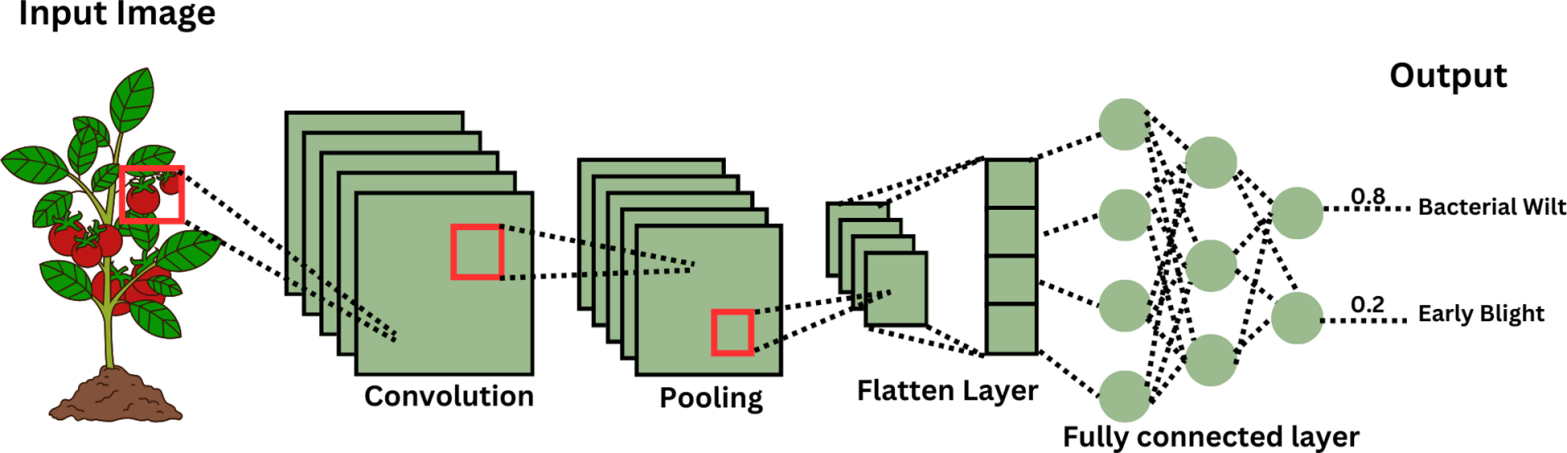
Structure of a Convolutional Neural Network (CNN).

CNNs formed the basis for plant disease identification from images, because of their ability to learn hierarchical spatial features automatically. Architectures like AlexNet, GoogLeNet, VGGNet, ResNet and Inception have consistently showed high performance in detecting plant and tomato-specific diseases. **Figure 3** illustrates the typical architecture and flow of CNNs in disease prediction. In the earliest studies, Mohanty et al. (2016) trained AlexNet and GoogLeNet on the PlantVillage dataset, achieving over 99% classification accuracy across 38 disease classes, including multiple tomato diseases. This pioneering work established the feasibility of using deep CNNs for automated plant pathology.

Building on this, Ferentinos (2018) assessed five CNN models (AlexNet, VGG, GoogLeNet, ResNet, and LeNet) using a large dataset containing 87,848 images of healthy and diseased plant leaf images, including tomato. The study found that classification accuracies, especially with AlexNet and ResNet, exceeded 99.53%. These results demonstrate the deep CNN’s robust learning ability when trained on large, well-curated datasets.

But, these models comprise tens of millions of parameters and rely on powerful GPUs or cloud-based infrastructure. For example, ResNet-50 has about 25.6 million parameters, which makes real-time inference on embedded or mobile phones, difficult. The deployment of AI models in remote or resource-constrained agricultural settings, where such infrastructure is unavailable, is limited by this high computational demand limits (Kamilaris and Prenafeta-Boldú, 2018).

In order to address this gap, researchers have switched to lightweight CNN architectures that retain accuracy while reducing complexity, an approach we explore next.

#### 3.1.3 Lightweight CNNs

Even though traditional CNN models perform well, their large model size and high computational demands make them unsuitable for low-power device deployment, particularly in remote agricultural regions. In order to overcome these challenges, lightweight CNNs that balance efficiency and performance have been developed, allowing real-time inference on edge devices namely Raspberry Pi, NVIDIA Jetson Nano and smartphones. Sandler et al. (2018), developed MobileNetV2, which significantly reduces model parameters and computation by using depthwise separable convolutions and inverted residuals. The work by Ahmed et al., 2022, using a lightweight MobileNetV2-based architecture, achieved 99.3% accuracy, with a reduced computational footprint, on the Plant Village dataset (tomato). It is among the most popular models for mobile-based disease diagnosis due to its high efficiency and small memory footprint. Tan and Le (2019), introduced Efficient Net-BO, which uses a compound scaling technique to scale network depth, width and resolution. This helps to make better use of the model’s capacity without significantly adding complexity. Efficient Net-BO with an integrated attention module (Efficient NetB0-Attn) demonstrated high performance and efficiency appropriate for edge deployment, achieving 99.39% accuracy on plant disease classification (González-Briones et al., 2025).

**Figure 4:**
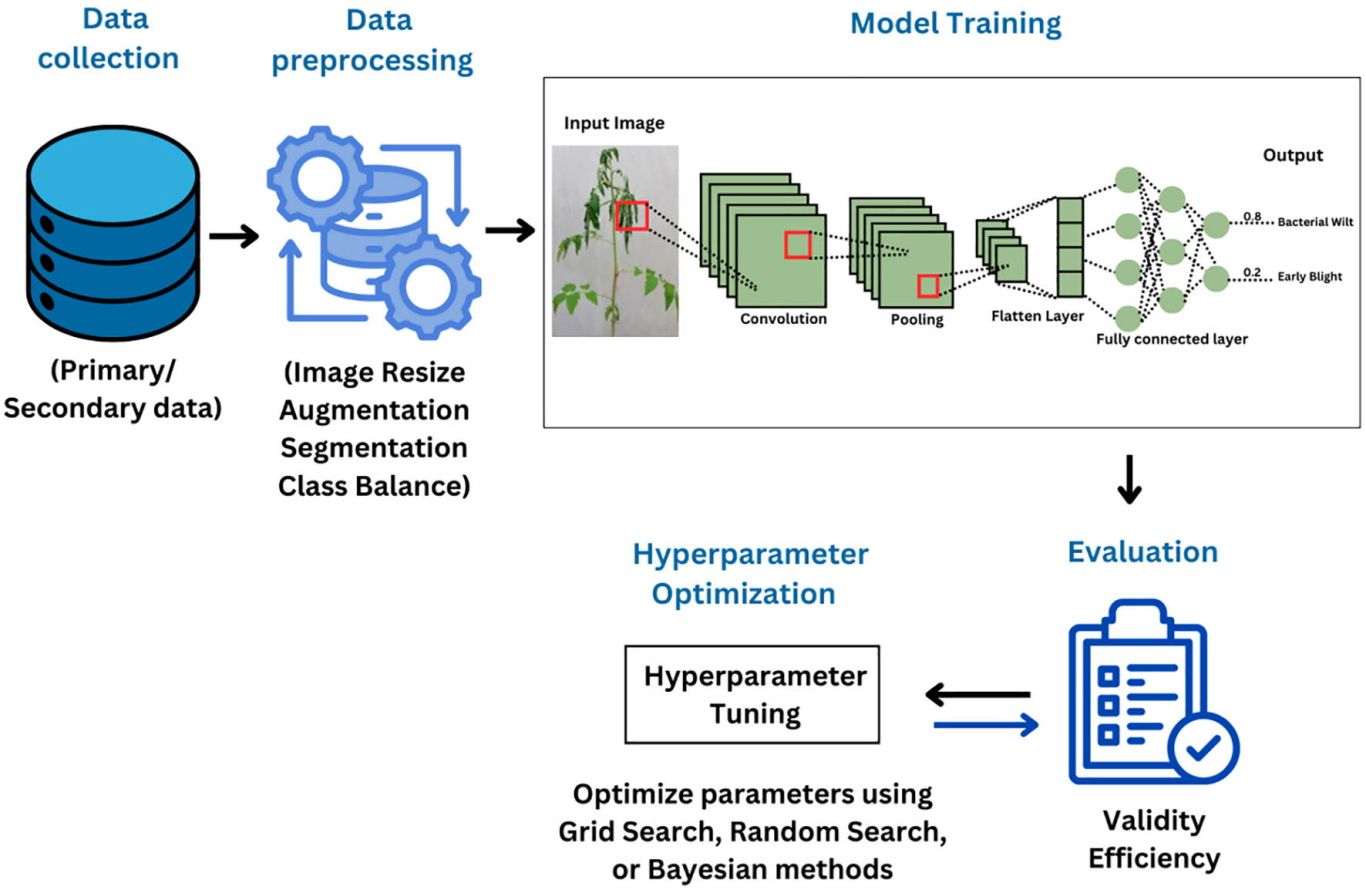
Overall workflow of deep learning-based tomato disease detection.

The efficacy of a siamese network-based lightweight framework for edge-AI deployment was demonstrated by its 96.97% accuracy on the tomato subset of the Plant Village dataset with roughly 2.96 million parameters (Thuseethan et al., 2024). These lightweight models represent a key advance in expanding access to AI-based disease diagnostics, especially for smallholder farmers in regions with limited data availability. **Figure 4** illustrates the overall pipeline for DL based tomato disease detection.

#### 3.1.4 Transformer-based and hybrid architectures: the rise of attention mechanisms in plant disease diagnosis

While CNNs are extremely effective in extracting local spatial features, they usually fail to extract long-range dependencies and global context, particularly in complex field images where background noise and overlapping symptoms is common. To address these limitations, transformer-based architectures initially created for natural language processing were utilized for computer vision tasks, including plant disease classification.

Dosovitskiy et al. (2021), introduced the Vision Transformer (ViT), which uses a self-attention mechanism to process image patches as sequences, thereby capturing both local and global interactions without the need for convolutions. On a balanced diverse tomato dataset, a multispectral approach utilizing ViT-B16 produced strong overall performance with average scores of 83.3% accuracy, 90.1% precision, 90.75% recall and 89.5% Fl-score, demontrating the efficacy of Vision Transformers in plant disease detection (De Silva and Brown, 2023). However, it is challenging to operate on low-power edge devices due to the model’s large size (about 86 million parameters), which results in significant processing demands.

To address this, researchers have developed MobileViT, a lightweight hybrid architecture that combines the inductive learning of CNNs with global modeling capability of transformers. MobileViT introduces local-global fusion blocks, that allows the model to achieve accuracy while maintaining computational efficiency. Based on this concept, a compact Vision Transformer (PMVT), modeled after Mobile ViT, demonstrated its suitability for mobile and resource-constrained deployment by achieving 94.9% accuracy with just 5.06 million parameters on plant disease detection tasks (Li G. et al., 2023). In order to achieve a strong balance of generalization, inference speed and overall performance, hybrid architectures such as CoAtNet (Dai et al., 2021) and Conformer (Peng et al., 2021) combines convolutional modules in early stages with self-attention mechanisms in deeper layers. These models offer great potential for future agricultural AI applications, even if they haven’t been thoroughly tested on tomato-specific datasets. The primary deep learning architectures for tomato disease detection are compiled in **Table 3** which also highlights the dataset used, parameter size, accuracy, and edge deployment suitability. The Transformer-based and hybrid architectures constitute a paradigm shift in AI for agriculture, offering enhanced generalization, interpretability and stability in challenging real-world situations.

**Table 3:** Summary of deep learning models for tomato disease detection.

Model	Architecture type	Parameters (Millions)	Accuracy (%)	Dataset	Edge deployability	Key insight	Reference
ViT-B16	Vision Transformer	~86	83.3 (Acc), 90.1 (Prec), 90.75 (Rec), 89.5 (F1)	Multispectral tomato dataset	No (cloud-only)	Very accurate but too heavy for real-time farm use	De Silva and Brown, 2023
PMVT	MobileViT-inspired Transformer	5.06	94.9	Plant disease dataset	Yes	Balances transformer power with mobile efficiency	Li G. et al., 2023
CoAtNet	Hybrid CNN + Transformer	Varies	Not tomato-specific	Generic datasets	Partial	Promising general model, needs crop-specific validation	Dai et al., 2021
Conformer	Hybrid CNN + Transformer	Varies	Not tomato-specific	Generic datasets	Partial	Good for mixed features, but untested on tomato datasets	Peng et al., 2021

#### 3.1.5 Multimodal and multi-source edge AI for tomato disease detection

Most lightweight deep learning models for tomato disease diagnosis is based on RGB imagery, recent research is going on in multimodal and multi-source sensing to improve robustness under real-field conditions. There are more challenges like illumination, partial occlusion, overlapping symptoms, and background clutter in image only systems. In order to address these issues, the developing edge AI techniques combine visual information with spectral, physiological, and environmental signals, which enables earlier and trusted disease detection.

Micro-near-infrared (micro NIR) sensors can now be combined with low-power controllers such as ESP32, as a result of the advancements in portable spectroscopy. This makes it possible for real-time reflectance measurements at wavelengths linked to water stress, chlorophyll degradation, and pathogen-induced biochemical changes. Research has demonstrated that NIR-RGB fusion can increase the accuracy of early disease detection by 5-12%, especially for fungal diseases, where pre-symptomatic physiological changes takes place before the development of visible lesions (Zhang et al., 2022; Park et al., 2023). These low-cost NIR modules (<10 mW power draw) can be embedded directly into edge-AI devices without significantly increasing latency.

In the same way, lightweight CNNs are being used in combination with chlorophyll fluorescence probes (Fv/Fin) to distinguish abiotic stress from bacterial or viral infection. For example, Ruiz-Gómez et al. (2021) demonstrated that TYLCV classification has been enhanced by combining chlorophyll fluorescence with RGB features under field conditions by increasing sensitivity to early chlorosis, which is sometimes challenging to detect visually.

Additionally, environmental sensors are also essential. It is found that the accuracy of disease prediction, for climate-dependent pathogen severity such as early blight and septoria, can be increased by combining soil moisture, canopy humidity, and temperature data with late-fusion neural architectures (Haque et al., 2022). These multimodal systems enhance model generalization across growth contexts and help in differentiating disease symptoms from temporary physiological stress.

Recent research explores lightweight multimodel fusion architectures that can operate on edge devices like Jetson Nano or ARM-based mobile processors, which goes beyond simple sensor integration. Techniques such as late fusion of CNN features, MobileNet-based hybrid networks, and attention-based fusion layers optimized for embedded systems (Koirala et al., 2023). When compared to image-only models, these models can show greater resistance in situations like uneven lighting and occlusion, while maintaining inference time suitable for real-time field deployment.

All of these advancements suggests that multimodel sensing can act as a new and crucial area for tomato disease detection, that is both useful and deployable. Moreover, edge-AI systems can overcome a number of drawbacks of RGB-based methods by utilizing spectral, physiological, and environmental data. This allows for earlier and more reliable diagnoses for precision agriculture applications. Multimodal and multi-source edge AI approaches for tomato disease detection is summarized in **Table 4**, which includes the data modalities used, edge platforms, and their advantages over RGB-only models.

**Table 4:** Summary of multimodal and multi-source Edge AI approaches for tomato disease detection.

Data modalities	Sensors/Inputs	Edge platform	Key advantage over RGB-only models	Representative studies
RGB + Micro-NIR	Micro-NIR reflectance, RGB images	ESP32, Jetson Nano	Improved early-stage disease detection; reduced sensitivity to lighting variation	Zhang et al., 2022; Park et al., 2023
RGB + Chlorophyll Fluorescence	Fv/Fm ratio, RGB images	Jetson Nano	Better discrimination between biotic and abiotic stress; improved TYLCV detection	Ruiz-Gómez et al., 2021
RGB + Environmental Data	Soil moisture, temperature, humidity	ARM-based edge devices	Enhanced robustness across climates; reduced false positives	Haque et al., 2022
Multimodal Fusion (RGB + Spectral + Environmental)	Combined visual, spectral, and microclimate data	Jetson Nano	Superior generalization under occlusion and uneven illumination	Koirala et al., 2023

### 3.2 Edge AI deployment strategies for tomato disease diagnosis

Achieving higher accuracy is only one aspect of deep learning in agriculture and the another important one is how easily these models can be used in environments with limited computing power. The majority of deep learning models are trained and assessed high performance GPUs or cloud platforms, but, the smallholder farmers in developing nations do not have access to such infrastructure. This has resulted in an increasing interest in edge-AI, where real-time inference is made possible by directly deploying on edge devices like drones, Raspberry Pi, smartphones or NVIDIA Jetson Nano, without the Internet dependency.

*Why Edge AI?*

Edge AI makes disease diagnosis to occur in local devices with the following advantages:

- Low latency: Predictions are generated within milliseconds, supporting timely decision-making in field conditions (Mazzia et al., 2020).
- Data privacy: Local processing of image data eliminates the need to send it to other servers. This lowers the risk of security and privacy issues associated with cloud-based data systems.
- Offline functionality: No Internet connection is required, which is important for remote or rural farms.
- Cost efficiency: Removes reliance on expensive cloud servers or network infrastructure.

Because of these advantages, edge deployment is an ideal solution for smart agricultural applications, especially in countries like India, where over 80% of farmers work on small landholdings with limited access to technology.

#### 3.2.1 Hardware platforms for field deployment

Deep learning has recently shifted from research labs to real-time agricultural conditions for tomato disease diagnosis. This change is largely influenced by edge-AI, which allows trained models to be installed directly on local hardware devices without the need for cloud-based inference. Raspberry Pi 4B, NVIDIA Jetson Nano and Android smartphones are the most common platforms used. Raspberry Pi 4B, a credit-card-sized microcomputer has a quad-core CPU and up to 8 GB of RAM, which balances both cost and performance. With the use of TensorFlow Lite, it easily supports lightweight models such as MobileNet, with inference speeds that are roughly 90% faster than the entire TensorFlow framework (Zuhair et al., 2023). Although it works well for simple tasks, its lack of GPU power may make it less effective for deeper models.

In contrast, real-time inference of moderate-sized CNNs such as ResNet and MobileNet is made possible by the NVIDIA Jetson Nano, which has a 128-core GPU and 4 GB of RAM. Because of this it is particularly useful in robotics and drone applications that need quick decisions and low latency (Tobiasz et al., 2023). Although it performs better, its increased cost and power consumption may be a drawback in farming areas with limited resources. Modern Android devices can run compressed deep learning models like MobileNetV2, with the help of frameworks like TensorFlow Lite or ONNX Runtime, especially those with adequate RAM and hardware acceleration (like mobile GPUs or NPUs). Depending on the model architecture and hardware optimization, these devices can achieve inference latencies of less than 500 milliseconds (Li Z. et al., 2023). However, in practical situations consistency may be impacted by hardware variability, thermal throttling and OS-level constraints. The features, capabilities and limitations are compiled in **Table 5** to help decide whether they are fit for different deployment situations.

**Table 5:** Comparison of edge AI hardware platforms for tomato disease detection.

Device	Specs	Supported models	Pros	Limitations	Best use case	Reference
Raspberry Pi 4B	4–8 GB RAM, Quad-core CPU	MobileNet (via TensorFlow Lite)	Low cost, widely available, open source; ~90% faster inference with TFLite	Limited GPU, slower for deep models	Low-cost farmer apps	Zuhair et al., 2023
NVIDIA Jetson Nano	128-core GPU, 4 GB RAM	ResNet, MobileNet	Real-time inference, GPU support, suitable for robotics/drone use	Higher cost, power-hungry	Drones, robotics	Tobiasz et al., 2023
Android Smartphone	4–12 GB RAM, Octa-core CPU + GPU/NPU	MobileNetV2 (TFLite, ONNX), SqueezeNet	Portable, farmer-accessible, <500 ms inference possible	Thermal throttling, hardware variability, OS fragmentation	Field-ready mobile apps	Li Z. et al., 2023

#### 3.2.2 Model optimization techniques

Model optimization is necessary for running deep learning models efficiently on edge devices, which usually have constrained memory and processing power. The most widely used optimization strategies are pruning, quantization and knowledge distillation.

Deeper architectures can be run on edge devices like Jetson Nano due to pruning techniques, as it drastically decreases the model size without affecting performance. For example, on devices like Jetson Nano, the FuPruner technique has shown efficient model compression with minimum loss of accuracy (Li et al., 2020).

Quantization decreases the size of the model and speeds up inference by reducing numerical precision typically from 32-bit floating-point weights into 8-bit integer format. Quantized models offer significant benefits in speed and energy economy, even though they lose very little accuracy (Jacob et al., 2018). They are therefore perfect for the deployment of micro-controllers and smartphones.

Knowledge distillation improves deployment efficiency by training a lightweight “student” model to simulate the output behavior of a more accurate, heavier “teacher” model. According to Hinton et al. (2015), this method can significantly reduce model complexity while maintaining a large portion of teacher’s accuracy.

The main goal, benefits and examples of each optimization method along with its limitation is listed in **Table 6** to provide a comparative summary.

**Table 6:** Summary of model optimization techniques for edge AI.

Technique	Purpose	Benefits	Limitation	Example use	Reference
Pruning	Remove unimportant weights to compress the model	Substantially reduces model size, preserves performance, enables deeper architectures on edge devices	May slightly reduce accuracy	FuPruner for compressed ResNet inference on Jetson Nano	Li et al., 2020
Quantization	Reduce precision of weights (FP32 → INT8)	Faster inference, smaller memory usage, improved energy efficiency	Needs post-training tuning, may cause minimal accuracy loss	MobileNet quantized for Android	Jacob et al., 2018
Knowledge Distillation	Train a smaller “student” model from a large, accurate “teacher” model	Retains much of teacher’s accuracy while reducing complexity	Requires a high-performing teacher model	MobileNetV2 distilled from ResNet	Hinton et al., 2015

#### 3.2.3 On-field deployment challenges

Edge AI has a lot of potential for smart agriculture, but there are numerous challenges to overcome before it can be used in real life settings. One major problem is dataset shift, which occurs when models were trained on ideal lab conditions (e.g., PlantVillage) are unable to generalize to field conditions because of variations in lighting, background clutter or disease stage (Kamilaris and Prenafeta-Boldú, 2018).

Inference instability caused by low-quality images such as incomplete, occluded or blurred images can reduce accuracy, which is an another major problem. Moreover, continuous usage of devices like Raspberry Pi in outdoors during hot weather might affect the performance of it due to battery limitations.

In order to address these issues, researchers are developing automated retraining processes, adaptive confidence thresholds and real-time image pre-processing that enables the model to be updated based on fresh field data. These methods are expected to be helpful in improving AI-based field diagnosis systems.

**Figure 5:**
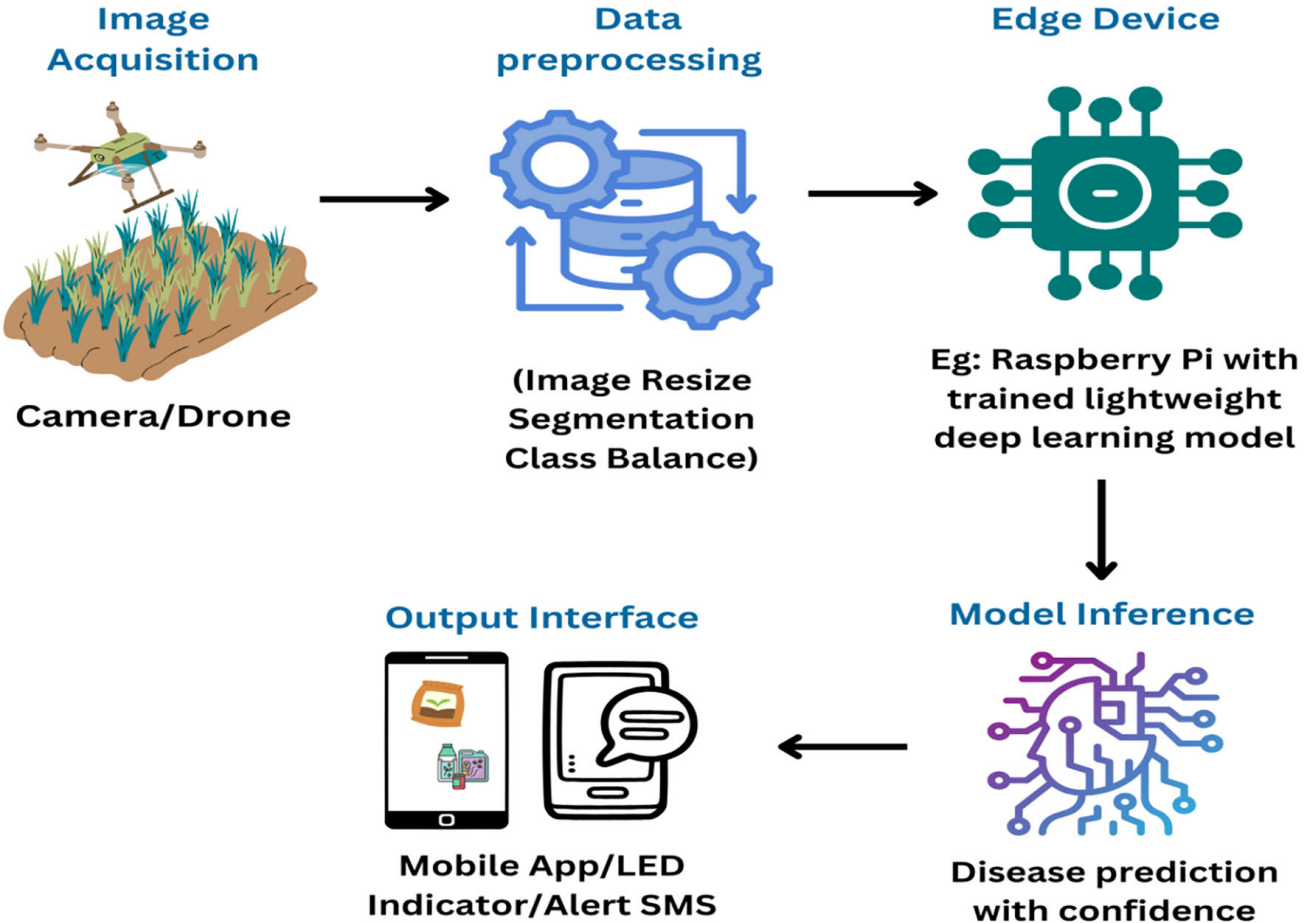
Edge AI process for detecting tomato plant diseases.

**Figure 5** shows a simplified flow that illustrates the normal inference process on an edge AI device.

### 3.3 Microbial biocontrol in tomato disease management

In addition to biotic stresses in tomato plants such as bacterial, viral and fungal infections, pathogen resistance to chemical pesticides is also increasing, posing environmental hazards. As a result of this concern, an environment friendly alternative, which is microbial biocontrol has gained attention that is used to suppress or outcompete plant pathogens and insect vectors.

#### 3.3.1 Mechanisms of action

Plant pathogens are suppressed by microbial biocontrol agents (MBAs) by using variety of mechanisms such as:

- Antibiosis: The synthesis of antimicrobial substances that either directly destroy or suppress pathogens.
- Mycoparasitism: The direct attack or destruction of pathogen hyphae and other structures.
- Induced Systemic Resistance, ISR (defensive response produced by beneficial microbes): Activation of plant immune responses against a broad range of pathogens.
- Competition for nutrients and space: Outcompeting harmful organisms within the rhizosphere or phyllosphere.
- Vector suppression: Certain microbes such as *Beauveria bassiana* target insect vectors responsible for transmitting viral pathogens.

Together these processes can produce a protective microbiome inside and around the tomato plant (Harman et al., 2004). The microbial biocontrol agents that are commonly used in tomato disease management with different modes of action are *Trichoderma*, *Bacillus*, *Pseudomonas*, *Beauveria*, and *Metarhizium*. For example, *Bacillus subtilis* competes for ecological niches by producing antibiotics whereas *Trichoderma harzianum* is well-known for its myco-parasitic activity and ability to trigger plant immune responses. The target pathogens and their representative biocontrol agents are shown in **Table 7**.

**Table 7:** Common microbial biocontrol agents used against pathogens in tomato plant.

Microbial agent	Target pathogens/Insects	Mechanism(s)	Field suitability	Key insight	Reference
*Trichoderma harzianum*	*Fusarium*, *Alternaria*, *Phytophthora*	Mycoparasitism, ISR, competition	High	Versatile, widely used, induces resistance	Yao et al., 2023
*Bacillus subtilis*	*Xanthomonas*, *Pseudomonas*, fungal pathogens	Antibiosis, competition, ISR	High	Effective as foliar spray, enhances photosynthesis	Chowdhury et al., 2015
*Pseudomonas fluorescens*	Soilborne fungi and bacterial wilt agents	ISR, antifungal compound production	Medium	Works better in root-zone treatments	Jia et al., 2023
*Beauveria bassiana*	Whiteflies, thrips (vectors of TYLCV, TSWV)	Entomopathogenic action	Medium	Useful against insect vectors like TYLCV carriers	Lacey et al., 2015
*Metarhizium anisopliae*	Root-knot nematodes, soil insects	Parasitism, colonization of insect cuticle	Medium	Niche role in soil-borne pest control	Harman et al., 2004

#### 3.3.2 Field applications and efficacy

Microbial biocontrol agents (MBAs) have been shown in multiple field studies to be extremely successful in managing a variety of plant diseases. For instance, *Trichoderma* species are mostly recognized for their role in the biological control of plant fungal and nematode diseases through mechanisms like mycoparasitism, competition, and induced systemic resistance (Yao et al., 2023). In the same way, foliar applications of *Bacillus subtilis* have demonstarted efficacy against bacterial spot (*Xanthomonas* spp.) and early blight (*Alternaria solani*), with treated plants having increased photosynthetic activity, improved leaf retention and enhanced fruit development (Chowdhury et al., 2015).

Apart from single-strain applications, microbial consortia with the combinations of two or more suitable biocontrol organisms are becoming more and more popular due to their synergy. By focusing on several infections or combining anti-fungal and insecticidal qualities, these formulations frequently offer broad-spectrum protection. For example, studies have demonstrated the synergistic antogonistic activity of *Bacillus*-*Pseudomonas* consortium, leading to improved control of fungal pathogen *Alternaria solani* (Jia et al., 2023). Depending on the pathogen’s infection pathway, these consortia are formulated as foliar sprays, soil drenches, root dips and seed coatings.

Additionally, field-based microbial trials are becoming more focused on evaluating the formulation’s stability, climate resilience and their effectiveness in different temperature and humidity levels. In order to improve soil health and plant resistance against diseases, recent research has investigated the use of organic amendments such as fermented, composted or fresh organic treatments in arable soils (van der Sloot et al., 2024).

Despite encouraging outcomes, soil microbiota, pH and crop type, all of these have a significant impact on field success. So, for scalability and adoption, region-specific strain selection and formulation is important. The suggested microbial agents, their doses, application methods and environmental factors are listed in **Table 8**.

**Table 8:** Multidimensional microbial recommendation matrix for tomato disease management under AI-guided decision support.

Disease/Pest pressure	Recommended microbial agent(s)	Dosage & formulation	Application method	Optimal growth stage	Environmental conditions (Temp, RH)	Reference
Tomato Yellow Leaf Curl Virus (TYLCV) (vector: *Bemisia tabaci*)	*Beauveria bassiana*, *Metarhizium anisopliae*	1×10^8^–1×10^9^ conidia/mL	Foliar spray targeting whiteflies	Vegetative, early flowering	Effective at 25–32 °C; RH > 60% enhances sporulation	Fargues et al., 2012; Wraight et al., 2020
Early blight (Alternaria solani)	*Trichoderma harzianum*, *Bacillus subtilis*	1–2 g/L WP or 1×10^8^ CFU/mL	Foliar spray + root drench	Seedling to vegetative	Best at 20–28 °C; moderate RH	Yao et al., 2023
Late blight (Phytophthora infestans)	*Trichoderma asperellum*	2–3 g/L WP	Preventive foliar application	Pre-flowering	Avoid applications during rainfall; RH 60–80%	Benítez et al., 2004
Bacterial wilt (Ralstonia solanacearum)	*Pseudomonas fluorescens*, *Bacillus amyloliquefaciens*	1×10^8^ CFU/mL soil drench	Soil drench near root zone	Transplanting + early vegetative	Effective at 25–30 °C; moist but not waterlogged soils	Jiao et al., 2021
Root-knot nematodes (Meloidogyne spp.)	*Paecilomyces lilacinus*, *Purpureocillium lilacinum*	2×10^8^ CFU/g granules	Soil incorporation around root zone	Seedling stage	22–30 °C; sandy loam soils preferred	Khan et al., 2019
Whitefly population surge (non-viral)	*Beauveria bassiana*	1×10^8^ conidia/mL	Direct foliar spray on vector hotspots	Any stage	RH > 65% improves infection	Wraight et al., 2020

#### 3.3.3 Controversies and challenges in microbial control

Microbial Biocontrol Agents (MBAs) are becoming more popular as environmentally friendly substitutes for chemical pesticides. But their efficacy in the field is still variable and sometimes inconsistent among research studies. These inconsistencies highlight several important challenges that must be addressed before microbial control can be consistently incorporated into precision disease management systems.

The foremost problem in the usage of microbial biocontrol is that its efficacy is sensitive to microclimatic factors. For example, *Bacillus subtilis* is the most commonly used foliar biocontrol agent but during high humidity, the antogonistic activity of it gets reduced. This is due to the inhibition of sporulation by excessive moisture and also it reduces persistance in leaves. In the same way, when *Trichoderma* species are applied in alkaline or low-organic matter soils, their efficiency is resduced. This shows that suitable environment is also essential for microbial biocontrol (Harman et al., 2004; Yao et al., 2023).

The next underlying problem is the strain adoption and colonization mechanism of the microorganisms. Because, most of the strains used for developing microbial consortia are grown in controlled environments like laboratory setups and tested in greenhouses. So, they fail to address the competition and stress conditions in agricultural fields. Field studies have demonstrated that microbial colonization is strongly dependent on soil microbiome composition, pH, temperature fluctuations and nutrient availability that leads to uneven disease control outcomes across regions (Jiao et al., 2021).

Another important conflict is that the chemical incompatibility between MBAs and certain classes of fungicides, insecticides and bactericides. For example, the viability and metabolic activity of *Bacillus* and *Trichoderma* is reduced by the combined usage of copper-based bactericides and broad-spectrum fungicides (Benítez et al., 2004). These interactions may unintentionally interfere with beneficial microbial colonization or decrease the biocontrol efficiency.

Further, the majority of MBAs have a short duration, which means they work best when they are used before or at the very early stages of pathogen infection. If application of MBAs is too late, then its efficacy can be greatly reduced, mostly affecting smallholder farming systems, because disease recognition often occurs late. It is common in quickly spreading diseases such as TYLCV or early blight. This limitation suggests the need for timely and precise application, which may be made possible by AI-based early detection systems.

At last, one of the most often constraint is the uneven field performance of the microbial consortia.

This unevenness is because of the complex interactions among environmental factors, crop genotype, native soil biota and agricultural management practices. These complications shows the importance of choosing region-specific strains, improve formulation stability and integrate digital decision support systems to increase acceptance and reliability.

All of these difficulties shows that although microbial biocontrol has a lot of potential, its practical application needs to be handled with considering all the external factors like biological interactions, environmental conditions etc. Understanding all these factors is crucial for developing strong and integrated control strategies that combine the advantages of AI-based diagnostics with microbial biocontrol strategies.

#### 3.3.4 Integration with AI-based early detection

Microbial biocontrol works best when it is applied right. Mostly biocontrol agents has to be used at the earlier stages of infection or pathogen colonization as they are defensive rather than beneficial. This makes them suitable for integration with AI-powered disease detection systems, particularly those capable of real-time, in-field diagnosis.

As discussed in Section 3, lightweight deep learning models such as MobileNet or ViT-lite can be deployed on edge devices (e.g., smartphones, Raspberry Pi, Jetson Nano) to detect early visual symptoms of foliar diseases like TYLCV or early blight. After detection, the system will suggest the suitable microbial agent, the application mode and its dosage. For example, if AI detects TYLCV symptoms in tomato then it will suggest the use of *Beauveria bassiana* to reduce whitefly populations before they reach outbreak levels.

According to the recent study by Rejeb et al. (2022), drones are being increasingly used in agriculture from monitoring crops to delivery of biocontrols due to their ability to provide focused treatments and more efficient resource management. This reduces the environmental impact and also minimizes the input cost for farmers. Moreover, geotagging of disease instances can help schedule repeated microbial applications or automatically create maps for agricultural extension services.

In order to provide a hybrid strategy for predicting disease outbreaks and optimizing the timing of microbial interventions, some advanced platforms are linking AI detection models with IoT-based soil and weather sensors. This level of linking is needed for closed-loop precision disease management, where AI not only diagnoses but also mitigates and monitors environment friendly treatment strategies.

### 3.4 Proposed framework

**Figure 6:**
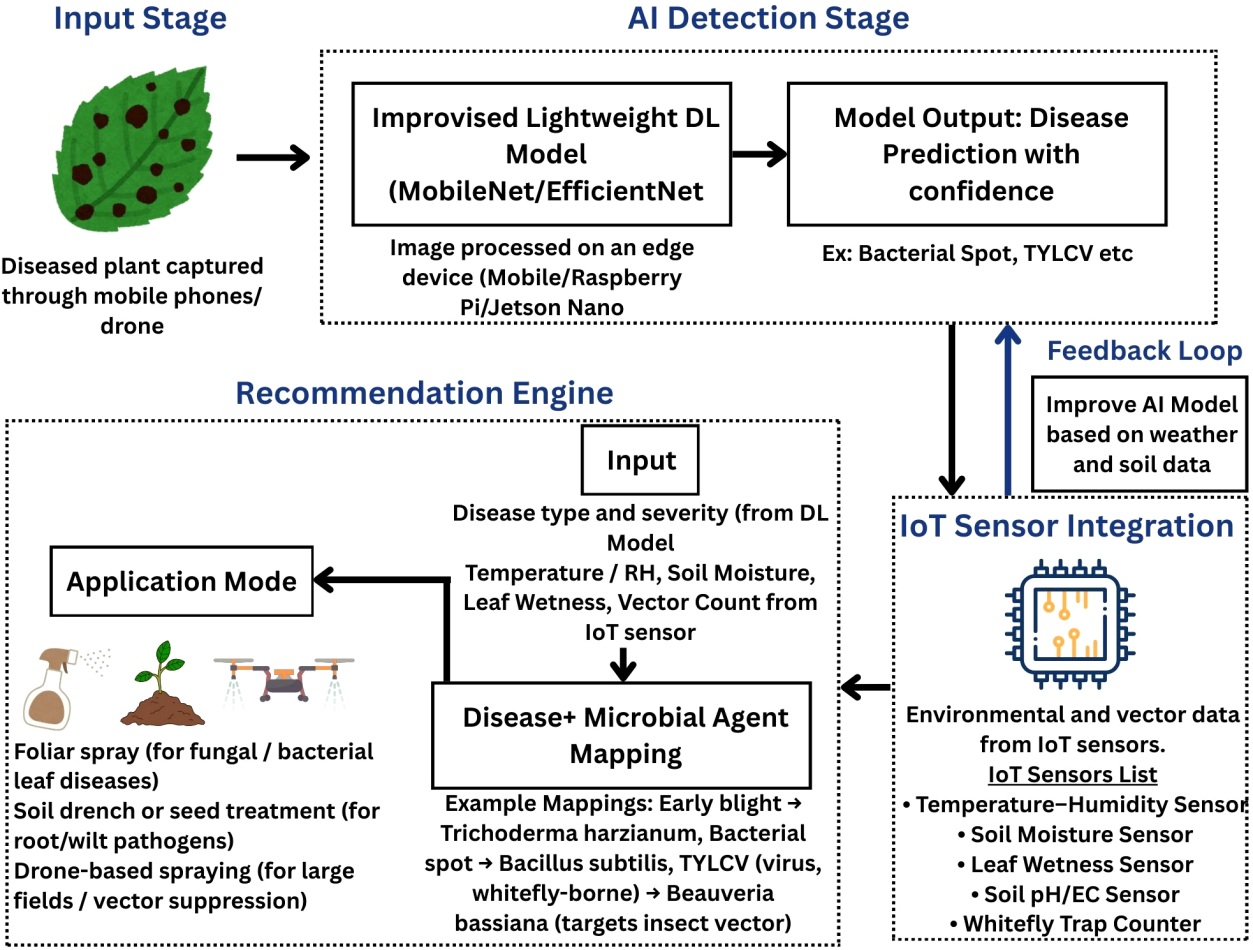
Proposed framework for edge AI–based tomato disease detection with microbial biocontrol strategies.

An integration framework that combines lightweight AI-based disease detection with IoT enabled sensing and microbial biocontrol strategies has been developed to address the gap between early disease diagnosis and actionable field management (**Figure 6**). This framework uses customized lightweight deep learning models like MobileNet and EfficientNet to process the tomato leaf images that are captured using mobile phones or drones and are processed on edge devices (eg. smartphones, Raspberry Pi, Jetson Nano). After analyzing, output of the model will be the type and severity of disease, which are then combined with microclimate and vector-pressure data obtained from IoT sensors (IoT sensor integration module). This includes, temperature–humidity measurements, soil moisture levels, leaf wetness status and whitefly trap counts.

Next comes the Recommendation Engine, which uses these AI-derived and IoT-derived parameters as inputs. This engine maps the identified disease to the proper microbial agents (e.g., *Bacillus subtilis* for bacterial spot, *Beauveria bassiana* for whitefly vectors, *Trichoderma harzianum* for early blight) and recommends the suitable application modes such as seed treatments, foliar sprays or soil drenches. In addition, recommendation engine also takes into account the environmental conditions and crop growth stages to adjust dosage and timing for maximizing the microbial efficacy under field conditions.

The IoT sensor integration module is connected to the AI detection stage through a feedback loop, which allows the system to continuously modify and enhance model performance as new field data is regularly updated. This closed loop framework (AI-IoT-Microbial bocontrol) helps farmers to take timely decisions before the disease outbreaks in a sustainable way. To the best of our understanding, no previous work has proposed an integrated architecture that directly connects edge AI–based tomato disease detection with microbial biocontrol recommendations in a real-time decision-support system.

## 4 Results and discussion

### 4.1 Dataset

All experiments were carried out in Google Colab with Python 3.12 TensorFlow 2.17 and PyTorch 2.4 frameworks. The colab environment’s NVIDIA T4 GPU was used for model training. Throughout the experiment, a fixed random seed (42) was applied for reproducibility. Using the Python tool kagglehub, The PlantVillage tomato leaf dataset was downloaded from Kaggle (Chaudhry, 2021). The subset containing 7,373 tomato leaf images was selected with an uneven distribution across four disease classes such as *Tomato Early blight* (800 images), *Tomato Late blight* (1,526 images), *Tomato Leaf Mold* (761 images), and *Tomato Yellow Leaf Curl Virus* (4,286 images). The dataset was split into 70% training, 15% validation, and 15% testing with the same fixed random seed (42) for consistency. Following the 70–15–15 split, the training, validation, and testing sets contained 5,160, 1,106, and 1,107 images respectively. **Table 9** presents the class-wise image counts for each split.

**Table 9:** Distribution of images in the curated PlantVillage tomato subset across training, validation, and testing sets.

Class	Total images	Training	Validation	Testing
Tomato Early blight	800	560	120	120
Tomato Late blight	1,526	1,068	229	229
Tomato Leaf Mold	761	532	114	115
Tomato Yellow Leaf Curl Virus	4,286	3,000	643	643
Total	7,373	5,160	1,106	1,107

### 4.2 Model performance

For experimental validation, two lightweight convolutional neural network architectures MobileNetV2 and EfficientNet-B0 were trained with a curated subset of the PlantVillage Tomato Leaf dataset. The dataset comprised four major tomato diseases prevalent in Tamil Nadu: *Early Blight, Late Blight, Leaf Mold*, and *Tomato Yellow Leaf Curl Virus (TYLCV).* All images were resized to 224 × 224 pixels and preprocessed according to the ImageNet normalization scheme specific to each model. Both architectures were started with ImageNet-pretrained weights using the *torchvision.models* API and fine-tuned for 10 epochs with Adam optimizer (learning rate = 1 × 10^−4^) and categorical cross-entropy loss. Data augmentation involved random horizontal flips and rotations up to 15°, while class weights were applied to reduce the influence of mild dataset imbalance. A batch size of 32 was used for training each model.

Results demonstrated that both models achieved exceptional classification accuracy on the test dataset. MobileNetV2 got an overall accuracy of approximately 99.9% with a macro-average F1-score of 0.99, while EfficientNet-B0 achieved a similar overall accuracy of 99.9% with a macro-average F1-score of 0.99. Although both the models have same accuracy, they differ by the number of parameters. MobileNetV2 required 2.26 million and EfficientNet-B0 has 4.05 million which is higher, confirming its superior computational efficiency and suitability of MobileNetV2 for real-time, resource-constrained deployment. The classification results of MobileNetV2 and EfficientNet-B0 is given in **Table 10**. Both the models achieved higher accuracy of 99.9% with MobileNetV2 showing slightly higher recall for Tomato Leaf Mold and better stability across classes.

**Table 10:** Performance comparison between MobileNetV2 and EfficientNet-B0 on the PlantVillage tomato leaf test dataset.

Model	Accuracy (%)	Macro avg precision	Macro avg recall	Macro Avg F1-Score	Epochs
MobileNetV2	100.0	0.99	0.99	0.99	10
EfficientNet-B0	100.0	0.99	0.99	0.99	10

**Figure 7:**
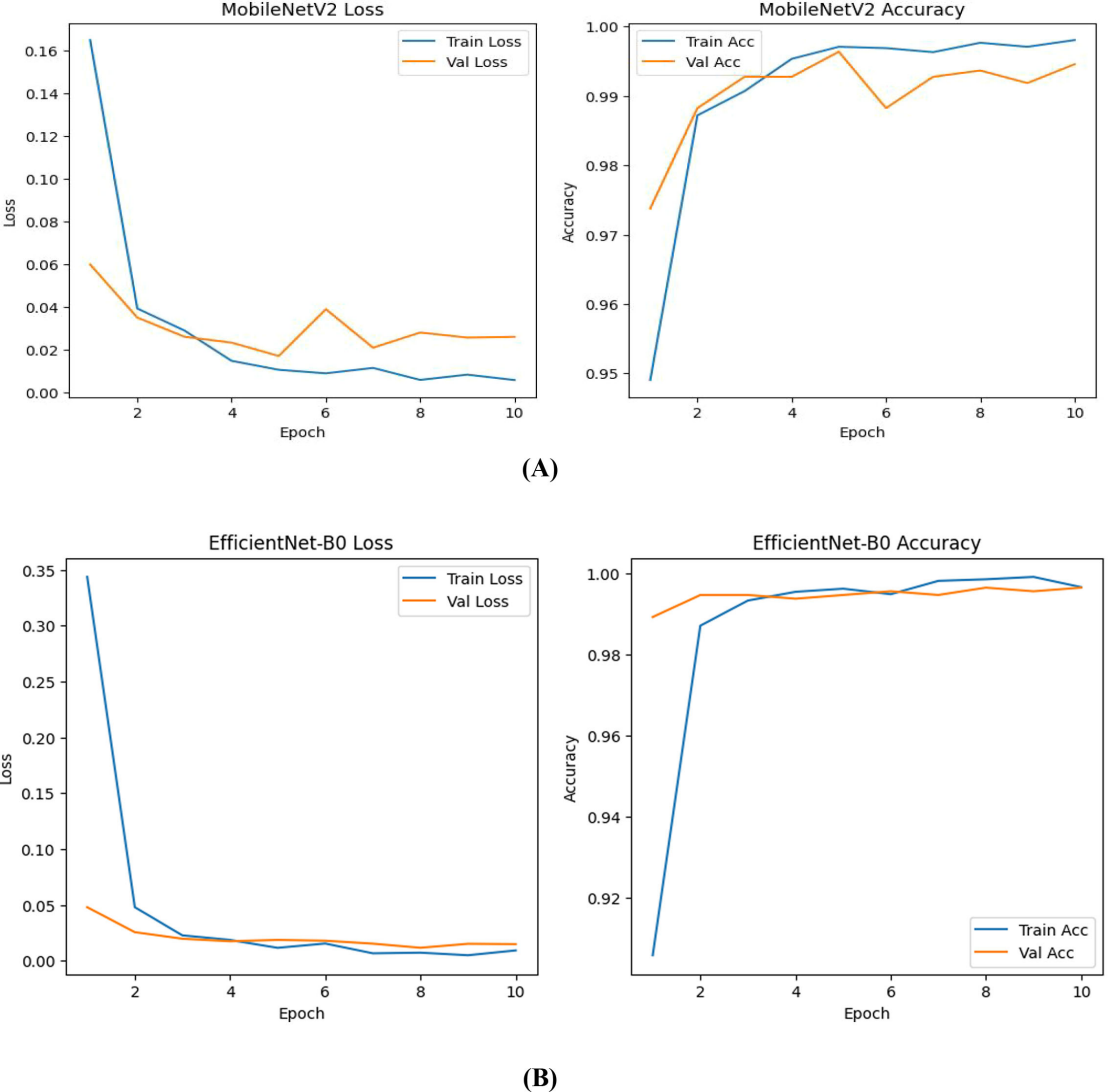
**(A)** Training and validation accuracy and loss curves of MobileNetV2 showing smooth convergence. **(B)** Training and validation accuracy and loss curves of EfficientNet-B0 showing smooth convergence.

**Figure 8:**
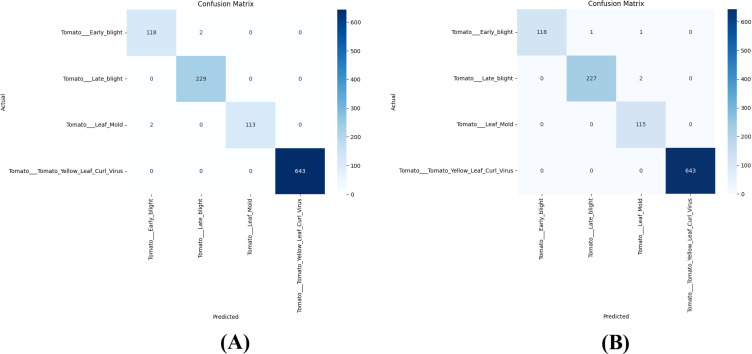
**(A)** Confusion matrix for MobileNetV2 showing perfect classification of all four tomato disease classes. **(B)** Confusion matrix for EfficientNet-B0 showing perfect classification of all four tomato disease classes.

The training and validation accuracy and loss curves of both models is shown in **Figure 7** demonstrating little overfitting and smooth convergence within 10 epochs. **Figure 8** shows the confusion matrices for each model, which verifies constant precision in every category.

Both models showed inference latencies of less than 100 milliseconds per image, indicating their suitability for tomato disease detection using edge-AI. From the findings it is confirmed that the lightweight models, specifically MobileNetV2 can achieve higher accuracy while retaining efficiency and generalization. This makes them appropriate for mobile and embedded agricultural systems.

### 4.3 Comparison with previous research studies

The outcome of this investigation aligns well with earlier studies on tomato leaf disease classification. Previous works by Ferentinos (2018); Ahmed et al. (2022), and Li G et al. (2023) have shown classification accuracies between 95% and 99% on the full PlantVillage dataset using deeper models such as VGG, GoogLeNet, and EfficientNet variants. Whereas, the present study also achieves a comparable accuracy using smaller sub dataset, less training epochs and fewer parameters. **Table 11** lists the comparative performance of both models used in current study and along with previous relevent studies. The class-wise precision and recall results shows equal performance across all four disease categories with only slight differences between *early blight* and *leaf mold*. This shows that transfer learning along with lightweight CNNs can efficiently match the performance of more complicated architectures and also maintains computational efficiency.

**Table 11:** Performance comparison between the present study and earlier existing works.

Author/Model	Dataset	Classes	Model params	Accuracy (%)	Notes
Ferentinos (2018)	PlantVillage (25 crops, 58 diseases)	58	AlexNet, GoogLeNet, VGG	99.5	Heavy models
Ahmed et al. (2022)	PlantVillage (Tomato)	10	MobileNetV2 (~2.3M)	95.6	Used full tomato dataset
This Work (2025)	PlantVillage (Tomato subset)	4 (Early Blight, Late Blight, Leaf Mold, TYLCV)	MobileNetV2 (2.26M)	99.9	Fast inference (100 ms), edge-deployable
This Work (2025)	PlantVillage (Tomato subset)	4 (Early Blight, Late Blight, Leaf Mold, TYLCV)	EfficientNetB0 (4.05M)	99.9	Underperformed on small, imbalanced dataset

Unlike large-scale research that depends mostly on generic datasets, the present work highlights region-specific validation by focusing only on tomato diseases commonly observed in Tamil Nadu. The AI model’s practical applicability to real time agricultural situations is improved by this targeted examination. From the above results, it is understood that transparency, adaptability and model efficiency are equally important for successful deployment of AI-based disease detection systems in field conditions, particularly in developing regions where computational resources are limited.

### 4.4 Field conditions and expected performance degradation in lightweight models

The strong basis for evaluating lightweight deep learning models was made possible by standard datasets like PlantVillage which also allowed for controlled model comparison. Recent studies including the current work uses advanced data augmentation techniques like illumination shifts, contrast variation, partial occlusion and background noise that simulates real field variability. This results in reduced model over-fitting. When going on for field deployment, these methods have been demonstrated to greatly enhance model generalization (Ferentinos, 2018; Too et al., 2019).

However, controlled datasets do not properly reflect the complexities in practical agricultural environments. Real field obtained images will have heterogeneous backgrounds, variable viewing, overlapping foliage, dew accumulation and early-stage symptoms that are frequently seen. According to studies by Too et al. (2019) and Agarwal et al. (2023), lightweight convolutional architectures may show decreased accuracy when directly assessed on unstructured field imagery, with performance degradation triggered more by environmental noise rather than model capacity.

Across the literature, the primary contributors to performance variation under field conditions include:

- Background complexity: soil, stems and debris introduce features similar to disease lesions;
- Occlusion and variable leaf geometry: Limits the visible symptomatic region;
- Early-stage disease expression: hard to differentiate from abiotic stress; and
- Imaging device heterogeneity: Includes differences in camera sensors and color calibration.

In order to overcome these challenges, lightweight pre-processing strategies compatible with edge deployment are suggested by numerous studies. Mobile-UNet and Fast-SCNN are the examples of background segmentation or leaf extraction modules that helps to decrease background interference and isolate the regions to be concentrated before classification (Agarwal et al., 2023). Likewise, Koirala et al. (2023) demonstrated that certain augmentation techniques like brightness variation, random occlusion masking and shadow modelling can enhance robustness without computational overhead. Further, predictions in complicated field conditions and uneven lighting can be stabilized by multimodel fusion techniques that combine spectral and environmental data with RGB images (Ruiz-Gómez et al., 2021).

All these findings reveal that, when lightweight models like MobileNetV2 and Efficient Net-B0 are combined with suitable augmentation and preprocessing techniques, are most suitable for edge-AI deployment. In order to enhance the shift from controlled benchmarks to operational agricultural systems, future research should be focused on verifying these methods using real time field datasets across different agro-climatic regions.

### 4.5 Implications for sustainable digital agriculture

The results of this experimental validation shows that combining lightweight deep learning models with sustainable disease management is practically feasible. The strong efficiency and accuracy of MobileNetV2 and EfficientNet-B0 shows the potential of edge-AI systems for offline and real-time disease detection, even in remote agricultural areas. These models can offer data-driven, environment friendly methods of managing pests and diseases when combined with microbial biocontrol strategies and decision-support systems.

Overall, this present work shows that lightweight CNN models based on transfer learning can attain superior performance while continuing to be deployable and interpretable on low power devices. By incorporating these AI-based disease diagnostic tools into precision agriculture, more scalable and resilient food production systems may develop.

## 5 Challenges and future prospects

Although there are a numerous advancements in AI-based disease diagnosis and microbial biocontrol strategies, their deployment is still hindered by many field level issues. The first and foremost challenge is quality and diversity of datasets, utilized for lightweight deep learning models. Most standard public datasets like PlantVillage has high-resolution images which is more suitable for training but they are captured in controlled environments with even lighting. These datasets lack field level variations such as uneven lighting, background noises, occluded leaves and overlapping symptoms. This difference between standard datasets and real-time scenarios has resulted in the performance gap in our study where model accuracy was lower than published standards due to dataset imbalance and restricted class variety. Therefore, in future research, priority should be given to the development of regionally varied, field-annotated datasets that records local disease expression and environmental factors and viral infections carried by insects.

The next challenge is the hardware deployment constraints. Though lightweight models like MobileNet shows high efficiency, their implementation on edge devices like NVIDIA Jetson Nano, Raspberry Pi, and Android smartphones are still constrained by computational power, battery capacity and thermal management. In high-resolution or real-time applications, inference delays can lower the reliability of diagnosis. Future research should address this issue by combining novel methods like TinyML and hardware-aware designs with model optimization methods including pruning, quantization, knowledge distillation, and neural architecture search (NAS) to produce powerful low-power AI models.

Although AI models are better at identifying diseases there is still little interaction of these models with microbial biocontrol strategies. To address this gap, we have proposed a framework (**Figure 6**) which combines AI diagnosis with microbial recommendations. But in order to achieve this synergy, microorganisms like *Trichoderma* spp, *Bacillus subtilis* and *Beauveria bassiana* must be used at the right time before the visible symptoms occur. To provide consistency, reliability and efficiency, microbial consortia must be customized for each region and field validated against different agro-climatic zones. Additional approach is combining these microbial agents with proper delivery systems like drone mounted dispensers or GPS sprayers.

Lastly, adoption by farmers still depends on trust and interpretability. It is common among farmers and extensive agents to accept the black-box predictions. Combining explainable AI (XAI) methods like Grad-CAM with mobile apps can increase transparency because they emphasize the leaf region that influenced the model prediction. Accessibility can be further improved by voice-assisted, multilingual and offline compatible interfaces in rural areas. In additon to technological advances, supportive institutional frameworks are needed to promote open datasets, standardize field evaluation protocols, and build farmer training programs.

In conclusion, the combination of sustainable microbial biocontrol strategies and lightweight AI models holds the key to manage tomato leaf diseases in the future. Moreover, in order to develop reliable, scalable and farmer-specific solutions, addressing dataset imbalance, hardware constraints, interpretability, and integration challenges are necessary that aligns with the aims of precision agriculture and sustainable crop protection.

## 6 Conclusion

New approaches for managing tomato diseases have been made possible by the convergence of artificial intelligence and sustainable crop protection. Deep learning approaches, particularly CNNs and transformer-based models have demonstrated great potential in automating leaf disease diagnosis from images, acting as a substitute for conventional labor-intensive methods. Moreover, real-time, on-field disease diagnosis on edge devices is made possible as a result of the advancements in lightweight architectures and other compression techniques. However, successful field deployment is still constrained by issues like model generalizability, dataset diversity and hardware constraints.

Simultaneously, microbial biocontrol agents are becoming more and more popular as environmentally accepted substitutes for synthetic pesticides. But their efficacy is relies heavily on timely and targeted application. This review highlights the unexplored potential of combining microbial biocontrol strategies with AI-based early detection to take focused and preventive actions. Development of field-representative datasets, enhancing model interpretability and creating closed loop system that connects diagnosis with smart decision support are all necessary for future advancements in this domain.

Our proposed framework in this study adds to this goal by combining microbial biocontrol techniques with AI-based early disease detection which goes a step forward than previous studies which addresses these components separately. This study provides an useful path for practical implementation by combining sustainable treatment and diagnosis in a single decision support system. This connection of intelligence and sustainability holds the key for managing tomato diseases and also opens the door to a new era of precision agriculture.
